# Promoting Critical Thinking and Digital Literacy in Nursing Students Through AI-Powered Podcasting: A Mixed-Methods Study

**DOI:** 10.3390/nursrep16040127

**Published:** 2026-04-10

**Authors:** Piyanut Xuto, Piyaporn Prasitwattanaseree, Tareewan Chaiboonruang, Lawitra Khiaokham, Nittha Panjaruang, Pattarada Chalermliamthong, Piyawan Sritawan

**Affiliations:** Faculty of Nursing, Chiang Mai University, 110/406 Intawaroros rd., Suthep, Muang, Chiang Mai 50200, Thailand; piyaporn.p@cmu.ac.th (P.P.); tareewan.c@cmu.ac.th (T.C.); prueksalada.k@cmu.ac.th (L.K.); nittha.k@cmu.ac.th (N.P.); pattarada.ch@cmu.ac.th (P.C.); piyawan.nuntapong@cmu.ac.th (P.S.)

**Keywords:** artificial intelligence, critical thinking, digital literacy, nursing education, podcasting

## Abstract

**Background:** Nursing education faces challenges in developing critical thinking and digital literacy among Generation Z students, particularly in maternal–newborn care contexts where evidence-based practice is essential. **Objectives:** To evaluate the effectiveness of an AI-assisted podcasting intervention on critical thinking and digital literacy among nursing students and explore their experiences. **Methods:** A convergent mixed-methods design included 48 third-year nursing students who created educational podcasts using AI tools (Sci Space for literature search, Notebook LM for synthesis). Quantitative data were analyzed using paired *t*-tests; qualitative data from three focus groups (n = 15) underwent thematic analysis. **Results:** Critical thinking scores increased significantly from 89.71 (SD = 13.43) to 117.29 (SD = 9.94), (t = −13.332, *p* < 0.001). Digital literacy scores improved from 37.98 (SD = 5.84) to 46.94 (SD = 4.11), (t = −9.407, *p* < 0.001). Four themes emerged: transformation from anxiety to empowerment, AI as scaffold, distinct tool utility, and future clinical application. **Conclusions:** These findings suggest that AI-assisted podcasting has the potential to significantly enhance critical thinking and digital literacy among nursing students; however, results should be interpreted with caution given the pre–post design, single-institution sample, and use of researcher-developed instruments.

## 1. Introduction

Contemporary nursing education confronts unprecedented challenges in preparing Generation Z students for evidence-based practice in an increasingly digital healthcare landscape. These digitally native learners require pedagogical approaches that simultaneously develop critical thinking skills and digital competencies while engaging them through familiar media formats [1]. The integration of educational technology in nursing curricula has become imperative, yet traditional teaching methods often fail to cultivate the analytical and technological skills essential for 21st century nursing practice [2].

Critical thinking represents a cornerstone competency in nursing education, enabling students to analyze complex clinical situations, evaluate evidence, and make sound clinical judgments [2]. Simultaneously, digital literacy has emerged as an essential skill set, encompassing the ability to locate, evaluate, and synthesize digital information effectively [1,3]. In maternal–newborn nursing education, where evidence evolves rapidly and patient education demands culturally appropriate communication, these competencies become particularly crucial. However, nursing students often struggle to bridge theoretical knowledge with practical application, especially when navigating vast amounts of online health information [1].

Podcasting has gained recognition as an innovative pedagogical tool in health professions education, offering opportunities for active learning, knowledge synthesis, and creative expression [4,5]. Student-created podcasts promote deep engagement with content, requiring learners to research, synthesize, and communicate complex information in accessible formats [6]. Recent studies have demonstrated podcasts’ effectiveness in raising awareness about specific health conditions [7,8], and enhancing student learning outcomes [9]. However, the integration of artificial intelligence tools to scaffold the podcasting process represents an emerging frontier in nursing education [10].

Constructivist learning theory provides the theoretical foundation for this intervention, positing that learners actively construct knowledge through meaningful engagement with authentic tasks rather than passively receiving information [11,12,13]. Within this framework, the act of creating educational podcasts for postpartum mothers represents an authentic, socially situated learning task that requires students to critically evaluate evidence, synthesize diverse sources, and transform complex clinical knowledge into accessible formats—processes that deepen understanding, promote retention, and mirror the cognitive demands of evidence-based nursing practice [11]. Of particular relevance is Vygotsky’s concept of the Zone of Proximal Development (ZPD), which describes the space between what a learner can accomplish independently and what they can achieve with guided support [12]. Vygotsky proposed that learning is most effective when scaffolding—structured support provided by a more capable agent—enables the learner to operate within this zone, gradually internalizing skills until independent competence is achieved [12]. In the context of this intervention, AI tools functioned as technological scaffolds within the ZPD: Sci Space (Typeset Inc., San Francisco, CA, USA; https://scispace.com/search, accessed on 10 October 2025) supported students in accessing and critically appraising evidence beyond their independent search capacity, while Notebook LM (Google LLC, Mountain View, CA, USA; https://notebooklm.google/, accessed on 10 October 2025) facilitated synthesis at a level of complexity that would have been difficult to achieve unaided. Critically, this scaffolding was not passive, the human-in-the-loop model required students to actively evaluate, verify, and refine AI outputs, ensuring that cognitive engagement remained with the learner rather than being offloaded to the technology. This positions AI not as a replacement for thinking but as a dynamic scaffold that progressively recedes as students develop competence and confidence—consistent with the constructivist principle that authentic challenge, supported by appropriate scaffolding, is the engine of meaningful learning [11,13]. Active learning theory further supports this approach, emphasizing that students learn more effectively through engagement, participation, and reflection than through passive reception of information [14]. Recent empirical evidence supports this theoretical framing, demonstrating that AI tools improve critical thinking outcomes in nursing and health professions education most reliably when embedded within structured, guided pedagogies that actively preserve the learner’s evaluative and analytical role [15,16,17].

Growing empirical evidence indicates that the relationship between AI tools and critical thinking is complex and highly dependent on how AI is used. A systematic review of nine studies examining AI-enabled educational tools in undergraduate nursing education found that improvements in clinical reasoning were more frequently observed when AI was embedded within guided, scenario-based pedagogies rather than used independently, and cautioned that AI should be regarded as complementary cognitive support rather than a substitute for supervised clinical mentorship [15]. Within nursing education specifically, perceived usability of AI tools was found to be a significant predictor of critical thinking motivation among nursing students, while the study also warned that overreliance on AI may lead to cognitive offloading and reduced independent analytical engagement [16]. Similarly, in higher education more broadly, there are two distinct patterns of AI interaction: passive, AI-directed use in which students accepted outputs uncritically, and collaborative, AI-supported interaction in which students integrated AI responses with their own knowledge—with enhanced critical thinking observed only in the latter pattern, and only when AI was used with structured guidance [17]. Collectively, these findings underscore the importance of designing AI-integrated curricula that actively position technology as a scaffold for higher-order thinking, the principle that informed the pedagogical design of the present intervention.

In Thailand, the integration of AI into higher education has recently become a national policy priority. The Ministry of Higher Education, Science, Research and Innovation formally established guidelines requiring all higher education institutions to embed AI tools into their curricula, develop AI competency among faculty and administrative staff, and establish dedicated institutional units to support AI-related teaching and learning [18]. This policy context is particularly relevant to nursing education, where digital transformation has accelerated and where educators are increasingly expected to model responsible AI use for students entering technology-driven clinical environments. The present study, conducted at a nursing faculty within a Thai public university, was therefore implemented within an institutional and national framework that actively supports AI integration in teaching and learning.

Despite growing interest in both podcasting and AI in nursing education, limited empirical evidence exists regarding the combined impact of these innovations on critical thinking and digital literacy development, particularly in maternal–newborn nursing contexts. Furthermore, student perspectives on AI-assisted learning remain underexplored. This gap in knowledge necessitates rigorous investigation to inform evidence-based integration of these technologies into nursing curricula.

This study addresses this gap by examining an innovative pedagogical intervention in which nursing students created educational podcasts about postpartum and newborn care using AI tools to support evidence searching and content synthesis. By employing a mixed-methods design, this research provides both quantitative evidence of learning outcomes and qualitative insights into student experiences, offering comprehensive understanding of how AI-assisted podcasting influences nursing student development. Thus, the primary objective was to compare critical thinking and digital literacy scores among nursing students before and after participating in an AI-assisted podcasting intervention.

**H1.**  *Critical thinking scores will be significantly higher post-intervention than pre-intervention.*

**H2.**  *Digital literacy scores will be significantly higher post-intervention than pre-intervention. The secondary objective was to explore students lived experiences and perspectives regarding the use of AI tools in creating educational podcasts.*

## 2. Materials and Methods

### 2.1. Research Design

This study employed a convergent mixed-methods design, integrating quasi-experimental quantitative methods with descriptive phenomenological qualitative inquiry. This approach enabled triangulation of numerical outcomes with rich experiential data, providing comprehensive understanding of the intervention’s impact.

### 2.2. Setting and Sample

The study was conducted at a Faculty of Nursing, Chiang Mai University, Thailand during the 2025 academic year. Participants were third-year nursing students enrolled in Maternal and Newborn Nursing and Midwifery Practicum 1, a required course focusing on postpartum and newborn care.

For the quantitative component, the course *Maternal and Newborn Nursing and Midwifery Practicum 1* operates on a rotating clinical practicum structure, with three student groups enrolled sequentially within the same semester (Group 1: n = 23; Groups 2 and 3: n = 24 each; total cohort n = 71). Funding for this study was confirmed approximately two weeks prior to the semester commencement. Following notification of funding approval, the research team developed both instruments, conducted content validity assessment and pilot reliability testing, and submitted the study protocol for Institutional Review Board (IRB) approval. Given the time required to complete these preparatory steps, IRB approval was obtained after Group 1 had already commenced their rotation. To protect the integrity of informed consent procedures and to ensure that all participants completed both pre- and post-intervention assessments under identical conditions, Group 1 was therefore not invited to participate in the quantitative data collection phase. Groups 2 and 3 (n = 48) were invited to participate following IRB approval; all 48 students provided written informed consent and completed both assessments, yielding a participation rate of 100%. It is important to note that the AI-assisted podcasting intervention itself was implemented as part of regular course instruction from the beginning of the semester for all three groups, as it was embedded within the active learning curriculum. The exclusion of Group 1 from data collection thus reflects an ethical and procedural decision rather than differential exposure to the intervention.

**Figure 1:**
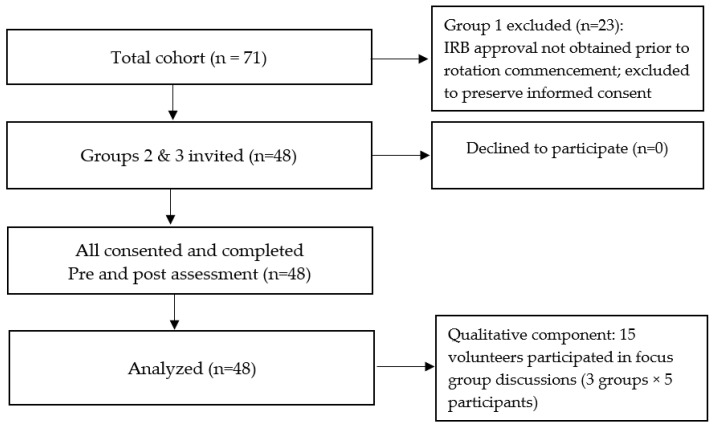
CONSORT-style participant flow diagram. IRB = Institutional Review Board.

For the quantitative component, 48 students participated in the intervention and completed pre- and post-assessments. For the qualitative component, 15 students volunteered to participate in focus group discussions, organized into three groups of five participants each, see in Figure 1. Purposive sampling ensured representation of diverse perspectives and experiences with the intervention.

Inclusion criteria: (1) Enrolled as a third-year nursing student in Maternal and Newborn Nursing and Midwifery Practicum 1; (2) provided written informed consent; and (3) completed both pre- and post-intervention assessments.

Exclusion criteria: (1) Enrolled in the first rotating practicum group (Group 1), as IRB approval had not yet been obtained at the time of their rotation commencement; (2) declined to participate or withdrew consent at any point during the study; and (3) did not complete either the pre-intervention or post-intervention assessment, resulting in incomplete data.

### 2.3. Instrument

Two researcher-developed instruments were used to assess critical thinking and digital literacy, both employing a five-point Likert scale (1 = strongly disagree to 5 = strongly agree). Total scores were computed by summing all items; no items were reverse-coded. Both instruments were administered as pre- and post-intervention assessments.

**Critical Thinking Scale**

The Critical Thinking Scale comprised 25 items across five subscales (five items each): Information Seeking, Analysis and Interpretation, Synthesis and Knowledge Organization, Problem-Solving and Decision-Making, and Self-Reflection. The scale was grounded on [19] critical thinking framework and contextualized to the AI-assisted podcast production environment in maternal and newborn nursing care, reflecting the higher-order cognitive demands of evidence synthesis, clinical decision-making, and reflective practice. Total scores ranged from 25 to 125, with higher scores indicating greater critical thinking ability. Representative items include: *“I can define clear and relevant keywords to search for evidence-based information on postpartum maternal care”* (Information Seeking); *“I can compare information from multiple sources to check for consistency or contradiction”* (Analysis and Interpretation); *“I can transform complex medical information into language easily understood by postpartum mothers”* (Synthesis); *“I can evaluate conflicting information from multiple sources and select the most credible content for presentation”* (Problem-Solving); and *“I can compare my own understanding before and after participating in the podcast production activity”* (Self-Reflection).

**Digital Literacy Scale**

The Digital Literacy Scale comprised 10 items across five subscales (two items each): Information Searching, Information Evaluation, Information Management, Technology Tool Usage, and Information Application. The scale was developed drawing on [20] three-dimensional digital literacy framework—encompassing technical, cognitive, and social-emotional dimensions of digital competence in educational contexts—and further informed by the Dig Comp 2.1 framework [21]. Items were contextualized to the AI-assisted podcast production environment, with Technology Tool Usage items specifically referencing the digital tools used in the intervention (Sci Space and Notebook LM). Total scores ranged from 10 to 50, with higher scores indicating greater digital literacy. Representative items include: *“I can use applications to efficiently search for evidence-based information online”* (Information Searching); *“I can distinguish between accurate and unreliable information on the internet”* (Information Evaluation); *“I can store and organize retrieved information systematically”* (Information Management); *“I can use software or applications related to research and media development for patient care”* (Technology Tool Usage); and *“I can apply information obtained from digital sources to evidence-based nursing practice”* (Information Application).

**Validity and Reliability**

Content validity for both instruments was established through review by a panel of three experts, all holding doctoral degrees (PhD) with established scholarly records in constructivist learning frameworks, AI and AI innovation in education, and active learning in nursing education, respectively. All three experts reviewed both instruments in their entirety, providing complementary domain-specific perspectives. Items were revised iteratively based on expert feedback until consensus was reached, and the content validity index was calculated at both item (I-CVI) and scale (S-CVI) levels following standard procedures [22]. Due to the constraints of funding approval timing and the compressed preparation period preceding the semester commencement, a separate pilot testing phase was not conducted prior to the main study. Internal consistency of both instruments was therefore evaluated using data from the study sample. The Critical Thinking Scale demonstrated excellent internal consistency (Cronbach’s α = 0.91), and the Digital Literacy Scale demonstrated good internal consistency (Cronbach’s α = 0.88), both exceeding the recommended threshold of 0.70 for research instruments [23]. The absence of a dedicated pilot testing phase is acknowledged as a limitation of the instrument development process; future studies should incorporate pilot testing with an independent sample to confirm psychometric stability prior to main data collection.

### 2.4. Intervention

Each participating group (Groups 2 and 3; n = 24 each) was subdivided into four clinical subgroups of six students, who rotated sequentially across three clinical settings within the course: the antenatal clinic (5 clinical days), the labor unit (10 clinical days), and the postpartum ward (5 clinical days). Clinical practice days ran Tuesday through Friday, with 2 additional days allocated to orientation and ward rounds at the commencement of the course and 1 day for the paper-based post-test at course completion, yielding a total practicum duration of approximately 8 weeks per group.

The AI-assisted podcasting intervention spanned the full 8-week practicum period and was structured around three sequential phases. The podcast product itself was anchored in postpartum maternal care, with all groups developing evidence-based podcast episodes specifically addressing the informational needs of postpartum mothers. However, the evidence-searching and critical appraisal skills developed in Phase 1 were intentionally designed to be transferable across clinical settings; students applied these search strategies to retrieve evidence relevant to their concurrent clinical rotation, whether in the antenatal clinic, labor unit, or postpartum ward, thereby reinforcing digital literacy and evidence-based practice competencies across the full practicum experience.

Phase 1 (Sci Space) involved structured evidence searching and critical appraisal of research literature, with students using Sci Space to identify and evaluate peer-reviewed sources relevant to postpartum maternal care while simultaneously applying search strategies within their concurrent clinical setting. Sci Space also supported translation of English-language articles into Thai, facilitating access to international evidence within the local clinical context.

Phase 2 (Notebook LM) involved AI-assisted synthesis and organization of retrieved evidence into a coherent podcast script, with Notebook LM used to integrate multiple sources into a structured narrative accessible to postpartum mothers. Phase 3 involved human-in-the-loop refinement, encompassing cultural appropriateness review, clinical accuracy checking by faculty, and final podcast production. All three phases were implemented progressively across the full 8-week Maternal and Newborn Nursing and Midwifery Practicum 1 course. Prior to commencing Phase 1, all students attended a 2 h orientation workshop in which faculty introduced both AI tools through hands-on practice exercises, ensuring baseline digital familiarity before independent use commenced.

### 2.5. Data Collection

Quantitative Data: Pre- and post-intervention scores on the Critical Thinking Scale and Digital Literacy Scale were collected from all 48 participants. The pre-test was administered on the first day of the course (orientation day), prior to any intervention exposure, to establish a genuine baseline measure of each participant’s critical thinking and digital literacy before the AI-assisted podcasting activities commenced [24]. The post-test was administered within one week of intervention completion, aligned with the course scheduling to maximize data completeness and minimize participant attrition [25]. Both the pre- and post-intervention questionnaires were distributed and completed electronically via a secure Google Form link, sent directly to students’ institutional email accounts. This method was selected to facilitate efficient data collection within the practicum schedule, ensure response completeness through required-field settings, and allow immediate data compilation for analysis. Paper copies were available for any students who preferred them, though all participants opted for the electronic format.

Qualitative Data: Three focus group discussions were conducted one week after intervention completion. Each 60 min session was facilitated by a trained researcher using a semi-structured interview guide. Questions explored students’ experiences using AI tools, perceived challenges and benefits, impact on learning processes, and perspectives on future applications. Focus groups were audio-recorded, transcribed verbatim in Thai, and translated to English by bilingual research team members. Transcripts were verified for accuracy through back-translation.

### 2.6. Data Analysis

Prior to analysis, the normality of score distributions were assessed using the Shapiro–Wilk test on the difference scores between pre- and post-intervention assessments, which is appropriate for paired-samples designs with n < 50 [11]. Results confirmed that both the critical thinking difference scores (W = 0.976, df = 48, *p* = 0.428) and digital literacy difference scores (W = 0.968, df = 48, *p* = 0.214) were normally distributed, satisfying the assumption for paired-samples *t*-tests.

Quantitative Analysis: Descriptive statistics (means, standard deviations) characterized critical thinking and digital literacy scores at both time points. Paired-samples *t*-tests assessed the significance of pre–post differences, with alpha set at 0.05. Effect sizes were calculated using Cohen’s d. Data analysis was conducted using statistical software.

Qualitative Analysis: Qualitative data were analyzed using reflexive thematic analysis following the six-phase framework described [26]: (1) familiarization with the data; (2) generating initial codes; (3) searching for themes; (4) reviewing themes; (5) defining and naming themes; and (6) producing the report. Two researchers independently conducted initial coding, identifying both semantic and latent patterns related to AI use, human-in-the-loop processes, and learning mechanisms. Codes were iteratively refined and organized into candidate subthemes and final themes through constant comparison. The research team met regularly to resolve discrepancies and reach consensus on theme structure. Credibility was strengthened through member checking, peer debriefing, and maintenance of a detailed audit trail documenting analytic decisions throughout the process.

Audio data from focus group discussions were transcribed verbatim in Thai by the research team and subsequently translated into English using Transkriptor (Transkriptor, Tallinn, Estonia; https://transkriptor.com/, accessed on 10 October 2025), an AI-assisted translation tool, to facilitate analysis and reporting. Translation accuracy was verified through back-translation by bilingual members of the research team. Subsequent theme development and refinement were conducted manually by the research team using Microsoft Word to organize, compare, and consolidate codes into candidate subthemes and final themes. Collaborative analysis sessions were held among researchers to reach consensus on theme definitions and boundaries. Throughout the process, Transkriptor was used solely to support translation of Thai focus group transcripts into English for reporting purposes; all analytical decisions, including the generation of codes, identification and development of subthemes and themes, refinement, and naming, were conducted entirely by the research team without AI assistance, ensuring full researcher ownership of the interpretive process, ensuring that the analysis adhered to the reflexive principle’s framework [26].

Mixed-Methods Integration: This study employed a convergent parallel mixed-methods design [27], in which quantitative and qualitative data were collected concurrently, analyzed independently as described above, and integrated at the interpretation stage. Integration occurred through a side-by-side comparison approach, whereby quantitative subscale outcomes were systematically compared with corresponding qualitative themes to assess convergence, complementarity, divergence, and expansion across data sources. This integration is presented as a joint display in the Results Section 3.

### 2.7. Ethical Considerations

The study was approved by the Institutional Review Board of the Faculty of Nursing, Chiang Mai University (No. 2568-EXP061). All participants provided written informed consent after receiving detailed information about the study’s purpose, procedures, risks, and benefits. Participation was voluntary, and students were assured that declining participation or withdrawing would not affect their academic standing. Confidentiality was maintained through use of participant codes, secure data storage, and reporting of aggregate findings only.

To minimize the potential for perceived coercion arising from the dual role of the research team as both course instructors and investigators, several procedural safeguards were implemented. Initial course orientation for Groups 2 and 3 was delivered by faculty staff who were not members of the research team. The research invitation, participant information sheet, informed consent form, and pre-test questionnaire were distributed at the end of the orientation session via Google Form, ensuring that students received study information in a context separated from direct researcher contact. Students were explicitly informed that participation was entirely voluntary and that declining or withdrawing would have no bearing on their academic standing or course evaluation. The AI-assisted podcasting intervention itself was embedded within the standard course curriculum and delivered to all three student groups regardless of research participation, meaning non-participation carried no academic disadvantage. Post-intervention data collectors occurred within the regular course schedule, further normalizing participation as part of course activity rather than as a special research obligation. These procedural arrangements were designed to minimize the perception of coercion and reduce the likelihood of social desirability bias in student responses. Nevertheless, the potential influence of the Hawthorne effect—whereby awareness of being observed as part of a study may itself motivate improved performance—cannot be entirely excluded and is acknowledged as a residual limitation of the study design.

## 3. Results

### 3.1. Quantitative Findings

All 48 students completed both pre- and post-intervention assessments. They had a mean age of 21.50 years (SD = 1.20) and a mean GPA of 3.25 (SD = 0.40). The majority were female (93.75%). Prior to the study, most students had used basic AI (ChatGPT 5.3 model) but lacked experience with research-specific tools like Sci Space (2.08%) or Notebook LM (2.08%) (Table 1).

Overall, the AI-assisted podcasting intervention was associated with statistically significant improvements in both Critical Thinking and Digital Literacy, with effect sizes that were notably large by conventional standards in educational research. Total Critical Thinking scores increased from a pre-intervention mean of 89.71 (SD = 13.43) to a post-intervention mean of 117.29 (SD = 9.94), t(47) = −13.332, *p* < 0.001, Cohen’s d = 2.36. Total Digital Literacy scores increased from 37.98 (SD = 5.84) to 46.94 (SD = 4.10), t(47) = −9.407, *p* < 0.001, Cohen’s d = 1.80. All subscale improvements were statistically significant at *p* < 0.001.

The most substantial gains in critical thinking were observed in the Analysis and Interpretation subscale (pre: M = 17.52, SD = 2.81; post: M = 23.65, SD = 2.25; d = 1.85), followed closely by Synthesis and Knowledge Organization (pre: M = 17.46, SD = 3.15; post: M = 23.33, SD = 2.27; d = 1.69) and Problem-Solving and Decision-Making (pre: M = 17.71, SD = 3.40; post: M = 23.42, SD = 2.43; d = 1.66). These three subscales, which correspond to the higher-order cognitive demands of Phases 2 and 3 of the intervention, evidence synthesis using Notebook LM and human-in-the-loop refinement, respectively, consistently showed the largest effect sizes, suggesting that the structured progression through AI-assisted synthesis and critical human review may have been particularly effective in developing complex analytical competencies. Reflective Thinking and Self-Assessment (d = 1.53) and Information Seeking and Evaluation (d = 1.52) also demonstrated large gains, though marginally smaller than the higher-order subscales.

Within the Digital Literacy Scale, the largest gain was observed in the Technology Tool Usage subscale (pre: M = 7.58, SD = 1.37; post: M = 9.44, SD = 0.87; d = 1.39), followed by Information Searching (pre: M = 7.31, SD = 1.29; post: M = 9.23, SD = 1.06; d = 1.17), Information Application (pre: M = 7.81, SD = 1.39; post: M = 9.46, SD = 0.92; d = 1.11), and Information Evaluation (pre: M = 7.42, SD = 1.32; post: M = 9.31, SD = 1.09; d = 1.10). The smallest—though still large—gain was observed in Information Management (pre: M = 7.85, SD = 1.57; post: M = 9.50, SD = 0.88; d = 0.96). The relatively stronger gains in Technology Tool Usage and Information Searching are consistent with the intervention’s explicit emphasis on hands-on use of Sci Space and Notebook LM throughout the 8-week practicum.

Regarding demographic characteristics, Spearman’s rank correlation indicated no statistically significant relationship between cumulative GPA and post-intervention Critical Thinking scores (ρ = 0.017, *p* = 0.907) or Digital Literacy scores (ρ = −0.032, *p* = 0.828), suggesting that intervention benefits were distributed equitably across students with varying levels of prior academic achievement. Gender subgroup analysis was not conducted given the highly disproportionate gender distribution (n = 3 male; n = 45 female), which would have rendered any between-group comparison statistically underpowered and uninterpretable.

The integrated findings indicate that AI-assisted podcasting functioned as a constructivist, HITL learning innovation. AI operated as a structured scaffold that prompted students to verify, critique, and refine AI-generated information. Through this iterative process, students demonstrated active curation, contextual judgment, digital navigation, and reflective decision-making, competencies that are essential in contemporary nursing practice.

### 3.2. Qualitative Findings

Thematic analysis of focus group data revealed four major themes that illuminated students’ experiences with AI-assisted podcasting and contextualized the quantitative improvements observed.

*Theme 1: Transformation from Anxiety to Empowerment.* Students described an emotional journey beginning with apprehension and culminating in confidence and enjoyment. Initially, many students felt overwhelmed by the prospect of creating professional educational content while navigating unfamiliar AI technologies. One student reflected, “At first, I thought it would be really hard and time-consuming. I wasn’t sure how to even start with these AI tools.” However, as students progressed through the intervention, anxiety transformed into empowerment. They discovered that AI tools made complex tasks more manageable and that the structured process built their confidence incrementally. “It turned out to be much easier than I thought, and actually fun,” one participant noted. Another student shared, “By the end, I felt proud of what we created. I realized I could do things I didn’t think I was capable of before.”

This transformation appeared closely linked to the scaffolded nature of the intervention, which broke the intimidating task of podcast creation into manageable phases. Students appreciated the gradual skill-building approach that allowed them to master each tool before integrating their capabilities.

*Theme 2: AI as Scaffold—The Human-in-the-Loop.* Students consistently emphasized that AI tools served as cognitive scaffolds rather than replacements for thinking. Contrary to concerns that AI might promote passive learning, students described how the intervention required active critical engagement. They positioned themselves as curators, evaluators, and editors rather than passive recipients of AI-generated content, “We had to check everything the AI suggested against what we learned in class and what actually happens in Thai hospitals”, one student explained. Students recognized that AI-generated content sometimes lacked cultural appropriateness or clinical nuance relevant to Thai contexts, “The AI gave us good starting points, but we had to verify everything and adapt it to our local practices”, another participant noted.

This human-in-the-loop approach appeared to enhance rather than diminished critical thinking. Students described carefully evaluating AI outputs, identifying inaccuracies or gaps, and making informed decisions about content inclusion, “It made us think more critically because we had to decide what to keep, what to change, and what to add”, one student reflected. The necessity of verifying AI content against local healthcare contexts particularly strengthened students’ analytical skills.

*Theme 3: Distinct Tool Utility—Complementary Strengths*. Students articulated clear distinctions between the two AI tools’ purposes and strengths. Sci Space was valued primarily for efficiency in the literature searching and its translation capabilities, which enabled students to access English-language research and comprehend it in Thai. “Sci Space saved us so much time finding recent research. The translation feature helped us understand complex English articles,” one participant shared. In contrast, Notebook LM excelled at synthesizing multiple sources and identifying connections across articles. Students appreciated how the tool helped them see patterns and themes across diverse literature, “Notebook LM helped us put all the pieces together and see the big picture”, a student explained. Another noted, “It showed us how different studies connected to each other, which we might have missed on our own.” Students recognized that the tools’ complementary strengths created a synergistic workflow. The efficiency gained from Sci Space’s search capabilities allowed more time for the deep synthesis facilitated by Notebook LM, while both tools freed cognitive resources for the critical refinement work in Phase 3. This recognition demonstrated students’ metacognitive awareness of how different technologies support distinct cognitive processes.

*Theme 4: Future Application—Bridging Classroom to Clinic*. Students envisioned practical applications of their newly developed skills beyond the classroom, particularly in patient education. They expressed enthusiasm about creating podcast-based educational materials for patients and families, distributed via QR codes in clinical settings, “We could make podcasts about postpartum care or newborn feeding and put QR codes in the postpartum unit”, one student suggested. This theme reflected students’ recognition that the skills developed through the intervention—evidence searching, synthesis, critical evaluation, and digital content creation—transfer directly to professional nursing practice. Students appreciated learning tools they could continue using throughout their careers, “These AI tools will be useful when we’re working as nurses and need to find evidence quickly or create patient education material”, a participant noted.

The focus on future application demonstrated that the intervention successfully bridged academic learning with professional practice, helping students envision themselves as evidence-based practitioners and patient educators. This forward-looking perspective suggested that the intervention cultivated not only immediate skills but also professional identity development.

### 3.3. Integration of Quantitative and Qualitative Findings

The qualitative themes provided rich contextualization for the quantitative improvements in critical thinking and digital literacy scores. The significant increase in critical thinking scores (Cohen’s d = 2.36) was illuminated by Theme 2 (AI as Scaffold), which revealed how the human-in-the-loop approach required constant evaluation, verification, and refinement of AI-generated content. Rather than accepting AI outputs passively, students engaged in higher-order thinking as they assessed accuracy, cultural appropriateness, and clinical relevance. The necessity of adapting content to local healthcare contexts particularly strengthened analytical and evaluative skills.

Similarly, the substantial improvement in digital literacy scores (Cohen’s d = 1.80) was contextualized by Themes 3 and 4. Students’ articulation of distinct tool utilities (Theme 3) demonstrated sophisticated understanding of how different technologies support specific information processes as a key component of digital literacy. Their ability to strategically select and employ appropriate tools for searching, synthesizing, and creating digital content reflected advanced digital competencies. Theme 4 further illustrated digital literacy development through students’ confidence in applying these skills to future professional contexts, including patient education via digital platforms.

The convergence between quantitative and qualitative findings strengthens confidence in the intervention’s effectiveness (Table 2). The numerical evidence of significant improvements in critical thinking and digital literacy is corroborated and enriched by students’ detailed descriptions of how they developed these competencies through active engagement with AI-assisted podcasting.

## 4. Discussion

This mixed-methods study provides preliminary evidence suggesting that AI-assisted podcasting may enhance critical thinking and digital literacy among nursing students while supporting active, engaged learning. These findings should be interpreted within the context of the methodological constraints outlined in Section 4.5. The findings challenge concerns that AI tools might undermine critical thinking, instead demonstrating that thoughtfully designed AI-integrated interventions can serve as powerful cognitive scaffolds that promote higher-order thinking skills.

### 4.1. Critical Thinking Development Through Human-in-the-Loop Learning

The substantial improvement in critical thinking scores (30.7% increase, Cohen’s d = 2.36) represents a noteworthy outcome that merits careful interpretation. This finding aligns with constructivist learning principles, which posit that authentic, complex tasks promote deeper cognitive engagement than passive learning activities [10]. The AI-assisted podcasting intervention required students to engage in multiple critical thinking processes: analyzing research literature, evaluating evidence quality, synthesizing diverse sources, assessing cultural appropriateness, and making informed decisions about content inclusion and presentation.

Critically, the qualitative findings revealed that AI tools did not diminish critical thinking but created new demands for analytical engagement. The human-in-the-loop approach positioned students as active evaluators of AI-generated content, requiring them to verify information against their clinical knowledge and local healthcare contexts. This finding address concerns raised by educators regarding AI’s potential to promote passive learning or academic dishonesty [1]. Rather than accepting AI outputs uncritically, students in this study engaged in metacognitive monitoring and evaluation—hallmarks of critical thinking [2].

The necessity of adapting AI-generated content to local healthcare contexts provided particularly rich opportunities for critical thinking development. Students could not simply accept Western-centric evidence but had to evaluate its applicability, identify necessary modifications, and integrate local clinical guidelines. This cultural translation process required sophisticated analytical skills and deep engagement with both evidence and context—precisely the type of critical thinking essential for evidence-based nursing practice in diverse settings.

### 4.2. Digital Literacy Enhancement in the AI Era

The significant improvement in digital literacy scores (23.6% increase, Cohen’s d = 1.80) reflects the intervention’s success in developing competencies essential for 21st-century nursing practice. This finding extends previous research on digital literacy in nursing education by demonstrating that AI-integrated interventions can accelerate digital competency development [1,3]. The intervention addressed multiple dimensions of digital literacy: information searching and retrieval (Phase 1 with Sci Space), critical evaluation of digital sources (throughout all phases), synthesis of digital information (Phase 2 with Notebook LM), and digital content creation (Phase 3). This comprehensive approach aligns with contemporary conceptualizations of digital literacy as encompassing not only technical skills but also critical evaluation and creative production capabilities [1].

Notably, the absence of a significant correlation between GPA and post-intervention outcomes (Critical Thinking: ρ = 0.017, *p* = 0.907; Digital Literacy: ρ = −0.032, *p* = 0.828) suggests that the intervention may have supported equitable learning gains across students with varying levels of prior academic achievement, an encouraging finding for the broader applicability of AI-assisted active learning in nursing education.

Students’ recognition of distinct tool utilities (Theme 3) demonstrated sophisticated digital literacy that extends beyond basic technology use to strategic tool selection. This metacognitive awareness—understanding which tools serve which purposes—represents advanced digital competency that will serve students throughout their professional careers as they navigate evolving technological landscapes.

### 4.3. Podcasting as Pedagogical Innovation

The effectiveness of podcasting as a learning modality, demonstrated in this study, corroborates growing evidence from health professions education. Previous research has shown that student-created podcasts enhance engagement and learning outcomes in pharmacy education [5], medical education [4], and nursing education focused on specific health conditions [7,8]. This study extends this evidence base by demonstrating podcasting’s effectiveness in maternal–newborn nursing education and by integrating AI tools into the podcast creation process.

The multimodal nature of podcasting requires research, writing, speaking, and audio production engages students in diverse cognitive processes that promote deep learning. Students must transform complex medical information into accessible narratives, a process that requires thorough understanding and synthesis [6]. The addition of AI tools to scaffold this process appears to have amplified learning outcomes by enabling students to access broader evidence bases and engage in more sophisticated synthesis while maintaining the cognitive demands of critical evaluation and refinement.

Students’ enthusiasm about using podcasts for patient education (Theme 4) suggests an important bridge between educational innovation and clinical practice. Previous research has demonstrated the effectiveness of multimedia patient education in maternal-child health contexts [11]. By learning to create evidence-based, culturally appropriate podcasts during their education, students develop skills directly transferable to patient education roles, potentially enhancing health literacy among the populations they serve.

### 4.4. Practical Implications for Nursing Education

The findings of this study offer concrete guidance for nurse educators seeking to integrate AI-assisted active learning into clinical practicum curricula. The three-phase human-in-the-loop (HITL) model demonstrated in this study represents a transferable pedagogical framework that can be adapted across nursing specialties and institutional contexts, provided that several structural and ethical elements are preserved in implementation.

First, regarding the phased structure, the sequential progression from evidence searching (Phase 1: Sci Space) through AI-assisted synthesis (Phase 2: Notebook LM) to human refinement and production (Phase 3) was central to the intervention’s effectiveness. This phased design mirrors the cognitive scaffolding principles of Vygotsky’s Zone of Proximal Development, progressively withdrawing structured support as students develop competence and confidence with AI tools. Faculty implementing similar interventions are advised to resist compressing the three phases into a single session or combining Phases 1 and 2, as the deliberate separation of evidence searching from synthesis is what requires students to engage critically with the literature before delegating organizational tasks to AI—a sequencing decision that specifically targets the Analysis and Interpretation and Synthesis subscales, which showed the largest effect sizes in this study (d = 1.85 and d = 1.69, respectively). A minimum of 6–8 weeks is recommended to allow sufficient time for iterative refinement across all three phases.

Second, explicit AI-ethics instruction should be incorporated as a foundational component prior to or during Phase 1, rather than treated as supplementary content. In this study, the 2 h pre-intervention orientation workshop introduced both AI tools through hands-on practice; future implementations should expand this workshop to include structured discussion of AI limitations, hallucination risks, intellectual property considerations, and the ethical responsibilities of healthcare professionals when using AI-generated content for patient education. Students should be explicitly taught to regard AI outputs as a starting point requiring human verification rather than a finished product—a disposition that underpins the human-in-the-loop model and is directly reflected in Theme 2 of the qualitative findings: The Primacy of Human Judgment in Curation and Contextualization. Faculty may consider using case examples of AI-generated health misinformation as discussion prompts to make the ethical stakes concrete and clinically relevant for nursing students.

Third, cultural appropriateness review should be formalized as a discrete, assessable step within Phase 3 rather than treated as an informal editing task. In the Thai nursing education context, this review encompassed checking that AI-generated podcast content accurately reflected local postpartum care practices, used culturally appropriate language accessible to Thai postpartum mothers, and avoided assumptions embedded in Western-centric health literature that may not translate to the Thai clinical environment. Faculty implementing this framework in other cultural or national contexts should similarly develop a context-specific cultural review checklist aligned with the health literacy needs and cultural norms of their target patient population. This step is particularly important when AI tools are trained predominantly on English-language datasets, as cultural and linguistic gaps in AI outputs may be subtle and require discipline-specific clinical knowledge to identify and correct—a task that is authentically nursing-specific and educationally valuable precisely because it cannot be delegated back to the AI.

Fourth, the podcast bank model—in which completed student-produced podcasts are archived with QR codes and made accessible to postpartum mothers via ward notice boards or discharge information packs—represents a sustainable, low-cost patient education resource that gives the intervention genuine clinical impact beyond the classroom. Faculty are encouraged to establish an institutional review process for podcast content before distribution to patients, involving a senior clinical nurse or midwife as a final content approver, to ensure that student-produced materials meet professional and safety standards before entering clinical use.

### 4.5. Limitations

Several methodological limitations should be considered when interpreting the findings of this study. First, the absence of a control group represents the most significant constraint on causal inference. Because all students enrolled in the course participated in the same required practicum, withholding the AI-assisted podcasting intervention from a subset of students was neither ethically nor pedagogically feasible. Consequently, the observed improvements in critical thinking and digital literacy scores cannot be attributed solely to the intervention, as natural course progression, maturation effects, and general clinical learning experiences occurring concurrently during the 8-week practicum may have contributed to the gains. Future studies should consider a waitlist control design or a comparison arm using non-AI podcasting to isolate intervention-specific effects.

Second, the available sample was constrained by a combination of structural and procedural factors. The course operates on a rotating clinical practicum schedule comprising three sequential student groups (total cohort n = 71). Funding approval was received approximately two weeks before the semester commenced, and the time required to develop and validate both instruments, conduct pilot reliability testing, and obtain IRB approval meant that data collection could not begin until after the first group (n = 23) had already started their rotation. To preserve the integrity of informed consent procedures, Group 1 was excluded from data collection, limiting the study sample to Groups 2 and 3 (n = 48). This constraint, while ethically necessary, reduced the overall sample size and should be acknowledged when considering the statistical power of the analyses reported.

Third, the single-institution design limits the transferability of findings to nursing programmes with different curricular structures, resource levels, institutional AI policies, or student demographics. Although the predominantly female sample (93.75%) reflects the broader gender distribution in Thai nursing education nationally, the homogeneity of the sample may limit generalizability to more diverse student populations.

Fourth, both instruments were researcher-developed and demonstrated acceptable internal consistency (Cronbach’s α = 0.91 and 0.88, respectively) and content validity; however, construct validity evidence beyond content validity index (CVI) is limited, as no confirmatory factor analysis has yet been conducted. The self-reported Likert-scale format also introduces the possibility of response shift bias, whereby participants may recalibrate their self-assessment standards following the intervention, potentially inflating pre–post score differences. This may partly account for the unusually large effect sizes observed (Cohen’s d = 2.36 and d = 1.80), which exceed typical benchmarks in educational research and should be interpreted as exploratory estimates requiring replication in larger, controlled trials. Of particular interpretive importance is the close alignment between instrument items and the specific tasks of the intervention—items directly referenced evidence searching, AI-assisted synthesis, and human-in-the-loop refinement, the same activities students practised throughout the eight weeks. This construct-intervention alignment means the scales were inherently more sensitive to change produced by this specific intervention than a general-purpose critical thinking or digital literacy measure would be, and this is likely a central contributor to the unusually large effect sizes observed. Future studies should employ validated, general-purpose instruments alongside any context-specific measures to allow direct comparison with the broader literature.

Fifth, the study did not include long-term follow-up assessment. It therefore remains unknown whether the gains in critical thinking and digital literacy observed immediately post-intervention are sustained over time or transferred to clinical practice settings. Future research incorporating delayed post-tests and objective performance measures would strengthen the evidence base considerably.

Sixth, the effect sizes observed (Cohen’s d = 2.36 for Critical Thinking; d = 1.80 for Digital Literacy) are unusually large by conventional educational research standards [28] and warrant critical interpretation. Four methodological factors are likely contributors: (1) construct-intervention alignment bias—instruments were developed specifically for this intervention context and are inherently more sensitive to it than general-purpose measures would be [25]; (2) the absence of a control group, meaning natural course progression cannot be separated from intervention-specific effects [24]; (3) the small and homogeneous sample, which reduces individual variation and may amplify group-level estimates [29]; and (4) response-shift bias, whereby participants may recalibrate self-assessment standards post-intervention, inflating pre–post differences [30]. These effect sizes should be interpreted as exploratory estimates requiring replication in larger, controlled, multi-site studies using externally validated instruments. Additionally, the study was conducted by academic staff who were directly involved in delivering the course and evaluating student performance. Despite assurances of voluntary participation and steps taken to maintain confidentiality, students may have perceived implicit pressure to participate or to respond positively, introducing social desirability bias that could have inflated self-reported scores. Future studies should consider recruiting participants through an independent third party and implementing data anonymization procedures prior to academic staff accessing results, to further minimise the potential for perceived coercion.

## 5. Conclusions

This study makes a novel empirical contribution as one of the first to examine the combined use of AI-assisted podcasting—integrating Sci Space for evidence searching and Notebook LM for synthesis—on domain-specific critical thinking and digital literacy outcomes in maternal and newborn nursing education. Statistically significant improvements were observed across all subscales of both outcome measures, with the largest gains in Analysis and Interpretation (d = 1.85) and Synthesis and Knowledge Organization (d = 1.69), suggesting that the phased, human-in-the-loop structure may be particularly effective in promoting higher-order cognitive skills. While these findings are promising, they should be interpreted with caution given the pre–post design without a control group, the single-institution sample, the researcher-developed instruments, and the unusually large effect sizes that warrant independent replication before definitive conclusions can be drawn.

The human-in-the-loop model demonstrated in this study, in which AI tools serve as cognitive scaffolds requiring constant student evaluation, verification, and contextualization rather than passive consumption, offers a concrete and transferable pedagogical framework for nurse educators seeking to integrate AI responsibly into clinical practicum curricula. Practically, the phased structure, explicit AI-ethics instruction, and cultural appropriateness review steps described in this study provide actionable implementation guidance applicable across nursing specialties and institutional contexts. Additionally, the podcast bank model, student-produced, QR-code-accessible episodes distributed to postpartum mothers as patient education resources, demonstrates how AI-assisted active learning can generate sustainable clinical value beyond the classroom.

Future research should address the methodological limitations of this study through rigorous control, multi-site designs incorporating validated instruments, objective performance measures, and long-term follow-up to assess retention and clinical transfer. Studies examining the applicability of the human-in-the-loop podcasting framework across diverse nursing specialties, student populations, and cultural contexts are particularly encouraged, as the findings from this single Thai nursing faculty, while promising, cannot yet be assumed to generalize broadly.

## Figures and Tables

**Table 1:** Demographic characteristics of participants.

Characteristics	Frequency (%)	Mean (SD)
Age (years)		21.50 (1.20)
GPA		3.25 (0.40)
Gender		
Female	45 (93.75)	
Male	3 (6.25)	
Experience with Artificial Intelligence		
Chat GPT	46 (95.84)	
Sci space	1 (2.08)	
Notebook LM	1 (2.08)	
Familiar with AI		
Moderate	41 (85.42)	
Fluently	7 (14.58)	
Podcast developed experience		
No	46 (95.84)	
Yes	2 (4.16)	

**Table 2:** Joint Display of Quantitative Subscale Outcomes and Qualitative Themes from the AI-Assisted Podcasting Intervention (n = 48).

Subscale	Pre-Test M (SD)	Post-Test M (SD)	t(47)	*p*	Cohen’s d	Qualitative Theme & Subtheme	Integrated Meta-Inference
Critical Thinking Scale							
Analysis & Interpretation	17.52 (2.81)	23.65 (2.25)	−12.816	<0.001	1.85	Theme 2: The Primacy of Human Judgment—*Critical Evaluation of AI Output*	Convergence: Largest CT gain aligns with students actively verifying, correcting, and reinterpreting AI outputs. AI stimulated rather than replaced analytical thinking.
Synthesis & Knowledge Organisation	17.46 (3.15)	23.33 (2.27)	−11.696	<0.001	1.69	Theme 2: The Primacy of Human Judgment—*Audience-Centric Refinement*	Convergence: Students’ qualitative accounts of translating complex evidence into accessible language for postpartum mothers directly correspond to gains in synthesis and knowledge organization.
Problem-Solving & Decision-Making	17.71 (3.40)	23.42 (2.43)	−11.520	<0.001	1.66	Theme 3: Transformative Pedagogical Outcomes—*Professional Confidence and Application*	Complementarity: Quantitative gains in problem-solving are extended by students’ reported confidence in advising patients using evidence-based information, reflecting emerging clinical decision-making readiness.
Reflective Thinking & Self-Assessment	18.63 (3.15)	23.77 (2.19)	−10.617	<0.001	1.53	Theme 3: Transformative Pedagogical Outcomes—*Development of Digital and Analytical Skills*	Complementarity: Self-reflection gains are supported by students’ recognition of their own skill development and their ability to articulate growth in digital and analytical competencies across the intervention.
Information Seeking & Evaluation	18.40 (2.65)	23.13 (2.20)	−10.525	<0.001	1.52	Theme 1: AI as an Efficiency Catalyst—*User Skill Barrier to Entry*	Convergence with divergence: Scores improved significantly; however, qualitative data reveal that keyword precision remained challenging, indicating information seeking competency developed unevenly across students.
CT Total	89.71 (13.43)	117.29 (9.94)	−13.332	<0.001	2.36		
Digital Literacy Scale							
Technology Tool Usage	7.58 (1.37)	9.44 (0.87)	−9.610	<0.001	1.39	Theme 1: AI as an Efficiency Catalyst—*Accelerated Research and Information Access*	Convergence: Largest DL gain corresponds to students’ descriptions of Sci Space and Notebook LM accelerating research workflows, confirming hands-on tool engagement drove measurable digital competency gains.
Information Searching	7.31 (1.29)	9.23 (1.06)	−8.121	<0.001	1.17	Theme 1: AI as an Efficiency Catalyst—*User Skill Barrier to Entry*	Convergence: Improvement in search scores is contextualized by students’ recognition that precise keyword strategies were critical and initially difficult, illustrating that the learning curve itself was a driver of skill development.
Information Application	7.81 (1.39)	9.46 (0.92)	−7.703	<0.001	1.11	Theme 3: Transformative Pedagogical Outcomes—*Professional Confidence and Application*	Complementarity: Gains in applying digital information to practice are extended by students’ reported readiness to use evidence and AI tools in future clinical settings, suggesting transfer beyond the immediate learning context.
Information Evaluation	7.42 (1.32)	9.31 (1.09)	−7.652	<0.001	1.10	Theme 2: The Primacy of Human Judgment—*Critical Evaluation of AI Output*	Convergence: Improvement in evaluating information quality aligns with qualitative evidence that students learned to critically scrutinize AI outputs, recognizing that human judgment remains essential even when AI assists retrieval.
Information Management	7.85 (1.57)	9.50 (0.88)	−6.631	<0.001	0.96	Theme 1: AI as an Efficiency Catalyst—*Accelerated Research and Information Access*	Expansion: The smallest DL effect size corresponds qualitatively to students prioritizing efficiency over systematic organization, identifying information management as an area for more explicit instructional emphasis in future implementations.
DL Total	37.98 (5.84)	46.94 (4.10)	−9.407	<0.001	1.80		
						Cross-theme divergence: Students described frustrations with slow AI processing times and the need for extensive revision of AI-generated content (Themes 1 & 2)	Divergence: Implementation barriers were evident qualitatively but not reflected in quantitative outcome scores, indicating that strong measurable gains coexisted with practical challenges in deploying AI tools. This finding has important implications for faculty support planning in future implementations.

## Data Availability

The research data can be downloaded at: https://drive.google.com/drive/folders/1SBFHn1ZTWoxC4JDqBhzcSoTXYihqhC7V?usp=sharing (accessed on 3 March 2026).

## Public Involvement Statement

No public involvement in any aspect of this research.

## Guidelines and Standards Statement

This manuscript was drafted against the Good Reporting of a Mixed Methods Study (GRAMMS) guideline for mixed methods research [27]. A complete list of reporting guidelines can be accessed via the EQUATOR network: https://www.equator-network.org/ (16 January 2026).

## Abbreviations

The following abbreviation is used in this manuscript:

AI	Artificial Intelligence.
